# Integrated network-based multiple computational analyses for identification of co-expressed candidate genes associated with neurological manifestations of COVID-19

**DOI:** 10.1038/s41598-022-21109-3

**Published:** 2022-10-13

**Authors:** Suvojit Hazra, Alok Ghosh Chaudhuri, Basant K. Tiwary, Nilkanta Chakrabarti

**Affiliations:** 1 CPEPA-UGC Centre for “Electro-Physiological and Neuro-Imaging Studies Including Mathematical Modelling”, University of Calcutta, Kolkata, West Bengal India; 2 Department of Physiology, University of Calcutta, Kolkata, West Bengal India; 3 Department of Physiology, Vidyasagar College, Kolkata, West Bengal India; 4 Department of Bioinformatics, School of Life Sciences, Pondicherry University, Pondicherry, India

**Keywords:** Computer modelling, Dynamic networks, Genetic interaction, Regulatory networks, Systems analysis, Systems biology, Computational science, Software, Statistics, Comorbidities, Neurological manifestations, Computational biology and bioinformatics, Cellular signalling networks, Computational models, Computational neuroscience, Functional clustering, Gene ontology, Gene regulatory networks, Genome informatics, Literature mining, Microarrays, Network topology, Statistical methods, Neuroscience, Computational neuroscience, Diseases of the nervous system, Molecular neuroscience

## Abstract

‘Tripartite network’ (TN) and ‘combined gene network’ (CGN) were constructed and their hub-bottleneck and driver nodes (44 genes) were evaluated as ‘*target genes*’ (TG) to identify 21 ‘*candidate genes*’ (CG) and their relationship with neurological manifestations of COVID-19. TN was developed using neurological symptoms of COVID-19 found in literature. Under query genes (TG of TN), co-expressed genes were identified using pair-wise mutual information to genes available in RNA-Seq autopsy data of frontal cortex of COVID-19 victims. CGN was constructed with genes selected from TN and co-expressed in COVID-19. TG and their connecting genes of respective networks underwent functional analyses through findings of their enrichment terms and pair-wise ‘semantic similarity scores’ (SSS). A new integrated ‘weighted harmonic mean score’ was formulated assimilating values of SSS and STRING-based ‘combined score’ of the selected TG-pairs, which provided CG-pairs with properties of CGs as co-expressed and ‘indispensable nodes’ in CGN. Finally, six pairs sharing seven ‘*prevalent* CGs’ (*ADAM10*, *ADAM17*, *AKT1*, *CTNNB1*, *ESR1*, *PIK3CA*, *FGFR1*) showed linkages with the phenotypes (a) directly under neurodegeneration, neurodevelopmental diseases, tumour/cancer and cellular signalling, and (b) indirectly through other CGs under behavioural/cognitive and motor dysfunctions. The pathophysiology of ‘*prevalent* CGs’ has been discussed to interpret neurological phenotypes of COVID-19.

## Introduction

The ‘coronavirus disease 2019’ (COVID-19) patients present common symptomatic features of dry cough, dyspnoea, fever, fatigue and myalgia followed by acute respiratory distress syndrome (ARDS) and multiorgan failure in an advanced stage^1^. COVID-19 is caused by severe acute respiratory syndrome coronavirus 2 (SARS-CoV-2), having positive single-stranded RNA as its genome^1,2^. The pathophysiological action of the virus always begins with the binding of spike proteins onto the angiotensin-converting enzyme 2 (ACE2) receptor proteins in the host cell membranes and expresses several phenotypic manifestations in human^2^. Human ACE2 receptors are constitutively expressed in different types of tissue cells in diverse regions of the brain^3^. COVID-19 causes structural/morphological changes in different areas of the brain^4^ and develops neurological and psychiatric symptoms^5^. Moreover, the SARS-CoV-2 infection in the brain culminates in inflammation of the meninges and perivascular space^6^.

The SARS-CoV-2 can enter the brain through three possible pathways via (1) the inflammatory supporting cells of the olfactory mucosa^6^, (2) the endothelial cells of the cerebral blood vessels^7,8^ and (3) the nerve terminals of the vagi in the respiratory^7,8^ and gastrointestinal tracts. SARS-CoV-2 is found to be present in the cerebrospinal fluid (CSF) of patients, suggesting the predominance of immunological damages over the viral replication in neurons^8^. Among the three pathways, the first one appears to be the most important, as a majority of the COVID-19 case reports suggest anosmia and ageusia/dysgeusia as the non-specific symptoms^9^. The clinical reports and the neuroimaging studies suggest that the cytokine storm and oxidative stress along with the reduction of GSH levels are two key mechanisms that can produce neurodegenerations in certain areas of the human brain^10,11^. It is speculated that COVID-19-related symptoms can together act as direct or indirect mediators of various neurodegenerative diseases including dementia, Alzheimer’s disease, and Parkinson’s disease, although the exact mechanism is still in debate.

The literature studies^12–15^ including the latest retrospective cohort study^13^ on 2,36,379 COVID-19 survivors indicate that > 30% of patients have neurological or psychiatric problems. The reports indicate that cognitive alterations (delirium with a combination of acute disturbances in attention, awareness and cognition, anxiety, sleep disorders), motor dysfunctions (dizziness, syncope, cerebellar ataxia, dysautonomia, seizure and epileptogenesis, tremors), cerebrovascular changes (cerebral ischaemia and infarct, stroke, focal ischemic necrosis, oedema, cerebral and subarachnoid haemorrhage, subdural haematoma, cerebral venous sinus thrombosis), cerebro-structural changes (meningitis/encephalitis, encephalopathy, necrotizing hemorrhagic encephalopathy, multifocal lesions in both cerebral hemispheres, leptomeningeal enhancement, myelitis, spinal cord myelitis, cranial neuropathy) are found in COVID-19 patients^12–15^. The symptoms related to disorders in the peripheral nervous system^13–15^ including muscle diseases (myopathy, muscle injury) and peripheral neuropathy/polyradiculopathy (viz. Guillain–Barré syndrome), may occur in certain cases.

In the present scenario, there is one bioinformatics-driven systems-level study using bipartite models of disease-gene, disease-disease, miRNA-gene and drug-protein interactions, which reveals that a variety of neurological symptoms including dementia, ataxia, encephalopathy and stroke along with their associated genes lined with multiple cellular functional pathways, can be therapeutically targeted by repurposed drugs or chemical compounds^16^. Additionally, a tripartite network modelling has been reported encompassing endocrine-disrupting chemicals (EDC), targeting proteins and diseases as the three types of nodes that decipher putative links between EDCs, COVID-19 severity and association to other diseases^17^. A systems-level modelling study^18^ has been conducted for the construction of a tripartite network of symptom-disease-gene to unravel the interplay between phenotype and genotype during disease conditions that are not limited to nervous system manifestation. Recent network-based findings of hub-bottleneck nodes for drug repurposing study report the involvements of several molecules associated with immunological systems (viz. cytokines e.g., TNFα, IL-1β,-6,-10 and chemokines e.g., CXCL8 and CCL2), growth factor function (e.g., VEGFA), cell-to-cell interaction (e.g., ICAM1), and signal transduction pathway (e.g., AKT1) with the neurological complication in COVID-19^19^.

In the present study, a novel approach has been introduced, for the first time to the best of our knowledge, to develop a model of predictive ‘*candidate genes*’ and their associations with neurological phenotypes of COVID-19. Initially, a tripartite network (TN) has been constructed using literature evidenced neurological symptoms of COVID-19 as input, whereby integrated weightage of symptoms and diseases are implied to get the most robust predictive genes for TN. Secondly, the predictive ‘*target genes*’ evaluated from TN have been considered as co-regulated in tissue and used as query genes to identify a set of co-expressed genes (CG) from RNA-Seq data of the frontal cortex of COVID-19 patients using pairwise mutual information (transcriptional gene–gene interaction from expression levels) to genes. The ‘combined gene network’ (CGN) has been constructed using genes selected in TN and co-expressed genes evaluated from RNA-Seq data of COVID-19. Both networks are analysed topologically and functionally to get ‘*candidate genes*’ and their connections with functional annotations to determine the putative molecular pathophysiology in the brain associated with COVID-19.

## Methods

**Figure 1:**
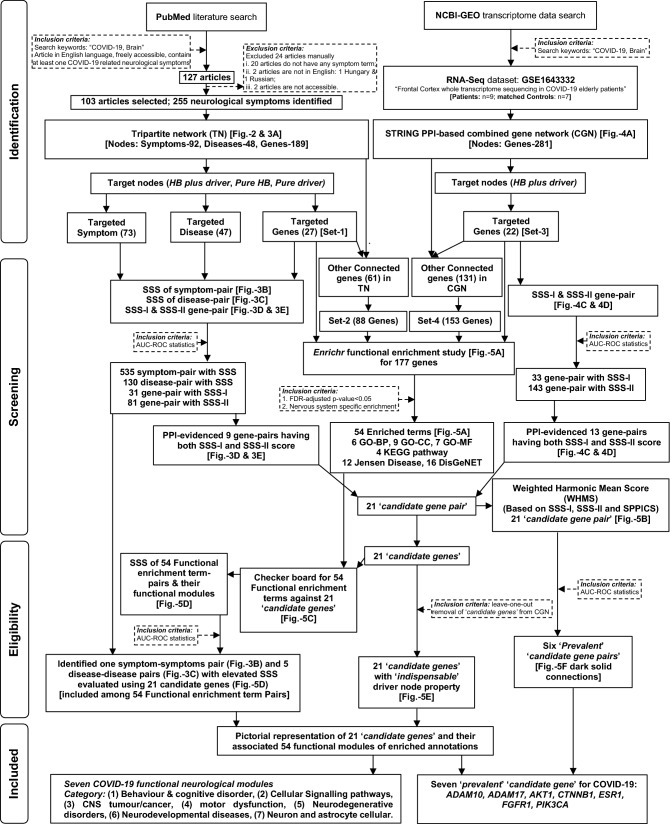
The flowchart of the stringent methodologies applied orderly and the results found in a systems-level analysis to identify ‘*candidate genes*’ and associated functional modules (symptoms/diseases) related to neurological manifestations of COVID-19.

The methodological approaches with inclusion and exclusion criteria applied in the present multiple computational analyses are documented in the flow diagram (Fig. 1). Briefly, this study was executed initially through literature search using keywords viz. ‘COVID-19, Brain’ as inclusion criteria. The literatures were selected based on exclusion criteria, and the ‘neurological symptoms/manifestations’ of COVID-19 patients were identified from these selected literatures. The exclusion of research articles (Table S1 in Supplementary File 1) and selection of ‘neurological symptoms/manifestations’ were curated manually (Table S2 in Supplementary File 1). Further, multiple steps with computational analyses had been introduced for the constructions of ‘Tripartite network’ (TN), integration of predicted genes found in TN with co-expressed genes in the brain of COVID-19 patients identified using the transcriptomic database to develop a ‘combined gene network’ (CGN) and finding of the ‘target genes’ followed by ‘candidate genes’ with their functional enrichments. Notably, the present study used several bioinformatics tools having respective methods and relevant citations (Supplementary File 2) mentioned in their respective web links.

### In silico modelling of the Tripartite Network (TN) for COVID-19

**Figure 2:**
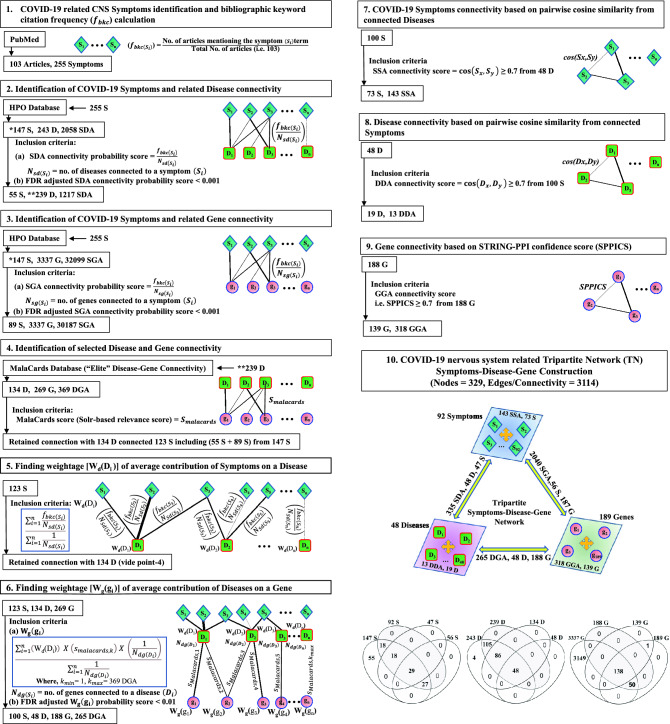
Study design for the construction of the tripartite network (TN) for COVID-19: Stepwise presentation of the construction of TN having symptoms, diseases and genes as nodes and their interactions as edges including inter-edges (viz., symptom-disease, symptom-gene, disease-gene) in point-1 to point-6 and intra-edges (viz. symptom-symptom, disease-disease, gene–gene) in Point-7 to point-9 of description. Point-1: Extraction of symptoms as terms associated with neurological disorders in COVID-19 from the PubMed bibliographic literature database and assigning a metric viz. bibliographic occurrence frequency (*f*_*bkc*_) to each term of symptom. Point-2 and Point-3: Extraction of the interactions between symptom-to-disease (point-2) and symptom-to-gene (point-3) from the HPO database using the symptom terms as queries and calculating their respective connectivity probability score followed by the selection of best-fitted connections using the statistical analysis of FDR-adjusted p < 0.001. Point-4: Extraction of the ‘Elite’ disease-gene connections with respective Solr-based relevance score (S_malacards_) from Malacards database using symptom-associated disease terms (found in point-2) as queries with respect to symptom-associated gene terms (found in point-3). Point-5 and Point-6: Implication of symptoms in connections of disease-genes by computing ‘weightage of average contribution’ of symptoms in diseases (W_d_(D_i_)) and that of diseases in genes (W_g_(g_i_)) to find the Elite’ Disease-gene connection considering co-occurrence of at least connections of one disease to one symptom and one gene terms in the network. The FDR-adjusted p < 0.01 was used to filter weighted-based disease-gene connections. Point-7 to Point-9: Finding intra-edges of nodes using cosine semantic similarity scores ≥ 0.7 for retaining symptom-symptom (point-7) and disease-disease (Point-8) pairs and, STRING confidence score ≥ 0.7 for retaining gene–gene pairs. These symptoms, diseases and genes are selected mathematically and statistically as described in point-6. Point-10: Integration of all inter- and intra-edges of symptoms and their allied diseases and genes to construct the TN (symptom-disease-gene) for COVID-19. The representative Venn diagrams indicate the stepwise changes in the number of nodes for the selection of elite symptoms, diseases and genes for the construction of TN.

The stepwise approach for construction of TN having nodes (symptom-disease-gene) and their connections (edges) developed by mathematical and statistical formulations is presented in the pictorial diagram (Fig. 2). Briefly, two bipartite networks (BN) of symptom-disease (step-2) and symptom-gene (step-3) were constructed where (a) terms/keywords of neurological symptoms related to COVID-19 collected by bibliographic literature search through PubMed portal (https://pubmed.ncbi.nlm.nih.gov/) were used as inputs (step-1) to retrieve (b) diseases and genes from the Human Phenotype Ontology (HPO, https://hpo.jax.org/) database. The symptoms were assigned by weightage (‘*bibliographic keyword citation frequency*’^20^; f_bkc(Si)_; Table S2 in Supplementary File 1) followed by finding their connectivity (N_sd(Si)_ or N_sg(Si)_; Table S3 in Supplementary File 1) probability scores to diseases (f_bkc(Si)_/N_sd(Si)_) and genes (f_bkc(Si)_/N_sg(Si)_) considering co-occurrence of at least one disease/gene connection to one symptom term following the principle of frequency of the co-occurrence of root node in the directed acyclic graph^21^. The connections between symptom-disease and symptom-gene were selected statistically (false discovery rate (FDR)-adjusted p < 0.001) in both BNs. Further, two BNs were linked through the implementation of “Elite” disease-gene interactions (*N*_*dg(di)*_; Table S4 in Supplementary File 1) using ‘Sorl’s relevance scores’ (S_*malacards*_) retrieved from Malacards (https://www.malacards.org/) database (step-4). Additionally, the disease-gene interactions were refined by two steps viz. (a) assigning integrated symptom-based weightage (W_d_(D_i_)) of diseases by calculating average ‘connection probability scores’ (f_bkc_(s_i_)/N_sd_(s_i_)) of symptoms to diseases (step-5) followed by (b) calculating integrated weightage (W_g_(g_i_)) of genes as disease-gene ‘integrated connection scores’ by multiplying W_d_(D_i_) with Malacards-scores (step-6) considering co-occurrence of connections of one disease to one symptom and one gene term in the network. The stringent connections in symptom-disease (step-2), symptom-gene (step-3) and disease-gene (step-6) were selected statistically (FDR-adjusted p < 0.001). The intra-edges of nodes were constructed using (a) ‘*cosine semantic similarity scores’* ≥ 0.70 for symptom-symptom and disease-disease pairs, considering each symptom node as a vector of connected diseases and vice versa^22^ (Table S5 in Supplementary File 1) and (b) ‘*STRING-PPI confidence score*’ (SPPICS) ≥ 0.70 (https://string-db.org/) for gene–gene pairs. TN was developed in Cytoscape software (http://www.cytoscape.org/).

### Finding out the co-expressed genes in the brain of COVID-19 patients using a database

The stepwise approach for finding co-expressed genes in the brain of COVID-19 patients using the NCBI-GEO database is presented in the pictorial diagram (step-1 to step-3 in Fig. 2). Briefly, the RNA-Seq data^23^ (NCBI-GEO accession ID: GSE164332) of the frontal cortex of the brain of COVID-19 victims (n = 9) and aged-matched healthy controls (n = 7), underwent analysis using *geneRecommender* algorithm (https://www.bioconductor.org/packages/release/bioc/html/geneRecommender.html) in R software and environment to identify co-expressed genes from the dataset against query genes, i.e., the genes having properties of both hub-bottlenecks and driver nodes evaluated in TN (vide point-4 in methodology). The input data set of the RNA-Seq samples was normalised followed by cross-validation with the leave-one-out method and genes were finally ranked based upon Spearman correlation with query genes using Z-score under *geneRecommender* analysis. The co-expressed genes were selected using the *minet* package (https://www.bioconductor.org/packages/release/bioc/html/minet.html) in R-language based on the algorithm for ARACNe (Algorithm for the Reconstruction of Accurate Cellular Networks) (https://rdrr.io/bioc/minet/man/aracne.html) assigning weights of (a) pairwise mutual information (transcriptional gene–gene interaction from expression levels) to genes as nodes and (b) empirical probability (entropy estimators) to its edge with a given threshold value for refining node-pairs.

### In silico modelling of ‘combined gene network’ (CGN) for COVID-19

A set of genes including genes as the output of *minet* analysis (co-expressed genes) and genes as the part of TN, were incorporated as a query in the STRING database using SPPICS > 0.60 as the threshold, to construct a PPI-based CGN (step-4 in Fig. 2). The study was further extended to construct CGNs using different ‘SPPICS’ viz. > 0.70, > 0.80 and > 0.90 as thresholds. The CGNs were developed in Cytoscape software.

### Topology analysis of networks to determine ‘hub’, ‘bottleneck’ and ‘driver’ nodes

The centrality measurements of networks were analysed using the *CentiScaPe* module (http://chianti.ucsd.edu/cyto_web/plugins/index.php) in Cytoscape software to determine ‘*hub*’ (high degree) and ‘*bottleneck*’ (high-betweenness/shortest-path) nodes, having higher scores than cut-off values i.e., respective average node degree and average node betweenness scores (for TN: 18.93 and 465.16; for CGN: (1) SPPICS > 0.60: 5.64 and 795.01, (2) SPPICS > 0.70: 4.43 and 644.21, (3) SPPICS > 0.80: 3.56 and 531.32, (4) SPPICS > 0.90: 3.17 and 406.16). These nodes are termed as the ‘*date-hubs*’ considering their properties as a higher level of the inter-modular connector to coordinate various functional complex modules in a complex biological network^24^.

The controllability measurements of networks were analysed with the identification of ‘*driver nodes*’ using the Minimum Driver node Set (MDS) algorithm from the *CytoCtrlAnalyser* module (https://apps.cytoscape.org/apps/cytoctrlanalyser) of Cytoscape software. The driver nodes control all nodes by receiving the input signals and provide the temporal/dynamic properties of a complex biological network. Driver nodes are classified into (a) ‘*indispensable*’ i.e., positive control factor, the removal of which increases the total number of driver nodes in the main network, (b) ‘*dispensable*’ i.e., negative control factor, the removal of which decreases the total driver nodes in the main network and (c) ‘*neutral*’ control factor, the removal of which does not change the total number of driver nodes in main network^25^, based on the ability of the nodes to control the main network. Therefore, the network control properties of selected nodes were assessed by the leave-one-out method (% changes of driver nodes after removal of the specific node). The ‘target nodes’ in networks were selected based on their ‘date-hub’ and ‘indispensable-driver’ properties, considering them as the disease candidates^25,26^.

### Gene ontology (GO) based pairwise semantic similarity score (SSS) measurement of ‘*target nodes*’

The pairwise functional associations of ‘*target nodes*’ were assessed by calculating SSS in Wang’s GO-BP (biological process) method using the *GOSemSim* package (http://bioconductor.org/packages/release/bioc/html/GOSemSim.html) in R-language using best-match average (BMA) combination strategy to get the results closer to human expectations^27^. The pairwise SSS measurements were performed in two separate functional packages namely, (a) ‘*mgeneSim*’ (https://rdrr.io/bioc/GOSemSim/man/mgeneSim.html) using a list of ‘*target genes*’ for assessment of ‘*direct association*’ with score designated as SSS-I, and (b) ‘*mclusterSim*’ (https://rdrr.io/bioc/GOSemSim/man/mclusterSim.html) using ‘gene-clusters’ (connected genes) against ‘*target nodes*’ of a network following the top-down approach^18^, for assessment of ‘indirect association’ with score designated as SSS-II. Further, the classifier statistics ROC-AUC was introduced using the *pROC* package (https://cran.r-project.org/web/packages/pROC/index.html) in R software and environment to filter out spurious pairwise SSSs for symptoms, diseases and genes considering the accuracy classification as excellent (0.9 < AUC < 1.0), good (0.8 < AUC < 0.9) and weak (AUC < 0.8). The optimal threshold for ROC based on the optimum F1-score and maximum accuracy was considered as a cut-off value for selected pairs of ‘target nodes’ as ‘candidate nodes’.

### Functional enrichment analysis of the sets of ‘*target genes*’ and their connected genes

The functional annotations across several resources including GO-terms (BP:‘biological process’, CC:‘cellular component’, MF:‘molecular function’), ‘KEGG biological pathways’ (https://www.genome.jp/kegg/pathway.html) and disease modules (DisGeNET, Jensen Disease) were analysed in the *Enrichr* web tool platform (https://maayanlab.cloud/Enrichr/) against the inputs of four different gene-sets viz. (1) Set-1: ‘*target genes*’ evaluated from TN, (2) Set-2: ‘*target genes*’ and their connected genes in TN, (3) Set-3: ‘*target genes*’ evaluated from CGN and (iv) Set-4: ‘*target gene*s’ and their connected genes from CGN. The Set-2 and Set-4 gene-sets were pondered to double-check the overrepresented functional annotation of the respective Set-1 and Set-3 gene-sets. The enriched results (functional annotations) associated with the nervous system were manually curated and selected based on FDR-adjusted p-value < 0.05 as significant terms, including the terms against the ‘*candidate genes*’. The pairwise SSS-II scores for statistically confident enriched terms were calculated using the *mclusterSim* function in the *GOSemSim* package using ‘gene-clusters’ (connected ‘*candidate genes*’). The classifier statistics ROC-AUC was used to find an accurate classification based on AUC values and select functionally associated Enrichr terms based on the optimal threshold for ROC as a cut-off of SSS-II scores.

### Formulation of integrated ‘weighted harmonic mean score’ (WHMS)

The integrated ‘weighted harmonic mean score’ (WHMS) was evaluated using harmonic mean of weightage scores for gene-pairs among ‘*target genes*’, which appeared to fulfil the criteria of having (a) three individual scores of SPPICS, SSS-I, SSS-II and (b) at least one score with a value above respective threshold (cut-off) level. The ‘*accuracy values of ROC*’ of SPPICS (W_SPPICS_), SSS-I (W_SSS-I_), SSS-II (W_SSS-II_) were applied as weightage for respective cases following the principle reported^28^ previously*.* The formula for integrated WHMS used in the study is given below.

$${\text{WHMS}} = { 3 } \times \, \left[ {\left( {{\text{W}}_{{{\text{SPPICS}}}} \times {\text{ SPPICS}}} \right)^{{ - {1}}} + \, \left( {{\text{W}}_{{{\text{SSS}} - {\text{I}}}} \times {\text{ SSS}} - {\text{I}}} \right)^{{ - {1}}} + \, \left( {{\text{W}}_{{{\text{SSS}} - {\text{II}}}} \times {\text{ SSS}} - {\text{II}}} \right)^{{ - {1}}} } \right]^{{ - {1}}}$$

The classifier statistics ROC-AUC and the optimal threshold for ROC as cut-off of WHMS were utilised to obtain the pairs *of prevalent* ‘*candidate genes*’ considering them as putative disease-associated genes.

## Results

The results found in our study were orderly documented in the flow diagram, with the findings of *prevalent* ‘*candidate genes*’ and their links with neurological functional modules in COVID-19 (Fig. 1). The 103 selected literature search identified the different statuses of COVID-19 patients and their 255 ‘neurological symptoms/manifestations’ (Table 1). Further analyses identified the connections of neurological symptoms/manifestations with co-expressed genes that were obtained from RNA-seq data.

**Table 1:** Summary of the facts reported in 103 literatures curated in PubMed database for finding the neurological symptoms of COVID-19 selected for the construction of TN.

Study type	Patients (% Males)	Timeline	PubMed ID of screened articles	Remarks on COVID-19 patients Period of CNS symptoms identified: Status of Patients
Case study (18)	4 (75%)	Jan–Apr, 2020	32314810, 32474220, 32457227, 32449057	During ICU hospitalisation: Died after 10–12 days
	1 (100%)	2020	32737799	During hospitalisation with COVID-19: Recovered during release
	1 (100%)	2020	32730234	During hospitalisation with COVID-19: Recovered during release
	2 (100%)	2020	32600350, 32409316	During hospitalisation with COVID-19: Recovered during release
	2 (50%)	2020	32615528, 32367205	During hospitalisation with COVID-19: Recovered during release
	1 (0%)	2020	32545925	During hospitalisation with COVID-19: Recovered during release
	1 (0%)	2020	32518103	During hospitalisation with COVID-19: Recovered during release
	1 (0%)	2020	32689590	During hospitalisation with COVID-19: Recovered during release
	3 (66.7%)	2020	32464585, 32430637, 32418288	During hospitalisation with COVID-19: Recovered during release
	2 (0%)	2020	32489724, 32586897	During hospitalisation with COVID-19: Recovered during release
Case series (6)	4 (50%)	2020	32679347	During hospitalisation with COVID-19: 3 patients with CNS problem during release
	2 (100%)	Mid-March, 2020	32462412	During hospitalisation with COVID-19: 1 died, 1 with long-term monitoring
	2 (50%)	2020	32464157	During hospitalisation with COVID-19: Recovered during release
	6 (83.3%)	Mar 16–Apr 5, 2020	32436105	During hospitalisation with COVID-19: 5 died, 1 with severe neurological deficits
	4 (25%)	2020	32360439	During hospitalisation with COVID-19: 3 died, 1 release
	2 (0%)	2020	32307298	During hospitalisation with COVID-19: 1 died, 1 release
Clinical cohort (16)	140 (71.4%)	May 3–May 5, 2020	32771053	During ICU hospitalisation with COVID-19: Not specified
	89 (61.8%)	Mar 23–May 23, 2020	32756734	During ICU hospitalisation with COVID-19: Not specified
	64 (67.2%)	Mar 6–Apr 9, 2020	32680942	During ICU hospitalisation with COVID-19: Not specified
	73 (65.8%)	Mar 23–May 7, 2020	32677875	During hospitalisation with COVID-19: Not specified
	86 (62.8%)	Feb 5–Apr 2, 2020	32754114	During ICU hospitalisation with COVID-19: Not specified
	9 (77.8%)	2020	32639679	During hospitalisation with COVID-19: Not specified
	43 (55.8%)	Apr 9–May 15, 2020	32637987	During hospitalisation with COVID-19: Not specified
	4 (50.0%)	Mar 1–May 8, 2020	32609336	During hospitalisation with COVID-19: Not specified
	50 (58.0%)	Mar 1–Apr 30, 2020	32570113	During hospitalisation with COVID-19: Not specified
	10 (80.0%)	Mar 1–Apr 15, 2020	32466736	During ICU hospitalisation with COVID-19: Not specified
	163	Feb–Mar, 2020	32467244	During hospitalisation with COVID-19: Not specified
	242 (62.0%)	Mar 1–Mar 31, 2020	32467191	During hospitalisation with early COVID-19: Not specified
	27 (74.1%)	Mar 1–Apr 14, 2020	32439651	During hospitalisation with COVID-19: Not specified
	454 (60.8%)	Mar 1–Apr 13, 2020	32447193	During hospitalisation with COVID-19: Not specified
	103 (57.3%)	Mar 30––Apr 24, 2020	32416289	During hospitalisation with COVID-19: Not specified
	58	Mar 3–Apr 3, 2020	32294339	During hospitalisation with COVID-19: Not specified
Systematic review (11)	205,938	2020	32730915	Not specified: Not specified
	765	2020	32725449	Not specified: Not specified
	116	2020	32603770	Long-term reported: Not specified
	36 (80.5%)	Mar 25–May 20, 2020	32653111	During COVID-19: Not specified
	9086 (45.2%)	Jan–June, 2020	32561222	Long-term reported: Not specified
	1454	Dec 2019-May 1, 2020	32574246	Not specified: Not specified
	1048 (50.4%)	Jan 1–Apr 10, 2020	32437679	Following COVID-19: Not specified
	235	2020	32422545	Not specified: Not specified
	-	Up to May 10, 2020	32490966	Not specified: Not specified
	4014	Jan 1–Apr 15, 2020	32345728	During COVID-19: Not specified
	765	Dec 1, 2019–Mar 26, 2020	32299017	During COVID-19: Not specified
Meta-analysis (1)	~ 4700	Feb 7-May 17, 2020	32529575	Not specified: Not specified
Narrative review, brief report, perspective, research article (51)	–	Feb 28–Aug 11, 2020	32776905, 32767055, 32758257, 32751841, 32729463, 32725545, 32720223, 32627524, 32683890, 32440692, 32672843, 32668062, 32628969, 32610334, 32655490, 32655489, 32491829, 32715280, 32587958, 32527073, 32581854, 32498691, 32492193, 32485101, 32486196, 32469504, 32474399, 32458193, 32574248, 32574247, 32442082, 32427468, 32424503, 32418055, 32427134, 32405259, 32405150, 32378030, 32417,235, 32417124, 32366614, 32643664, 32515379, 32352081, 32320066, 32343122, 32320211, 32266761, 32385132, 32104915, 32538857	During COVID-19 and long-term COVID, as indicative: Not specified

The facts indicate types of the studies in literatures, their publication timeline, sample size included in the studies, PubMed ID of the articles and status of the COVID-19 patients mentioned in the studies. The details of the literature PubMed ID with their full citation are presented in Table S1 under Supplementary File 1. The manually curated ‘neurological symptoms/manifestations’ from 103 selected literature are presented in Table S2 in Supplementary File 1.

### Construction of TN using symptoms, diseases and genes

**Figure 3:**
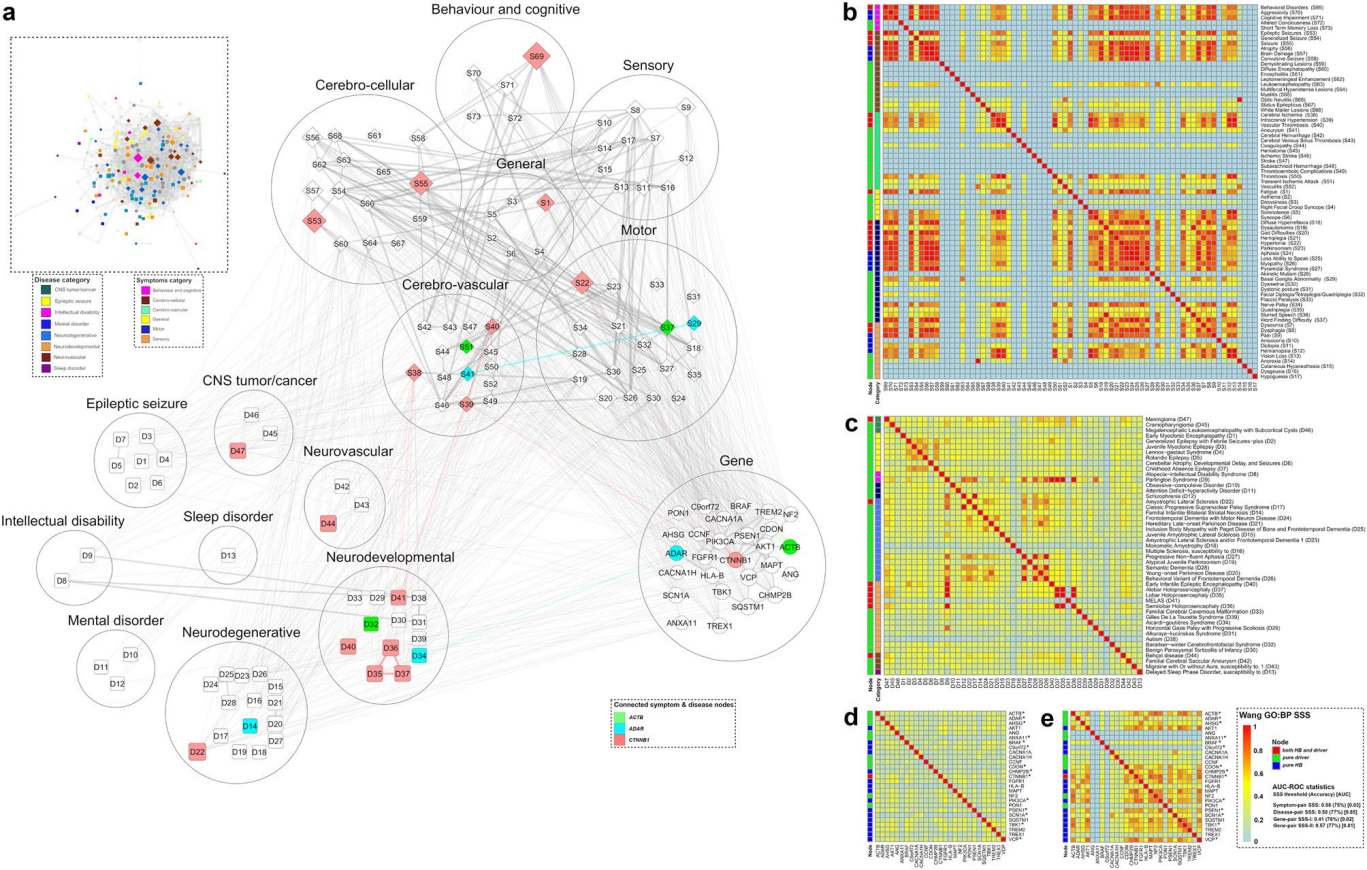
The model of ‘tripartite network’ (TN) and its ‘*target nodes*’ with their pairwise semantic similarity scores (SSS) for COVID-19: (**a**) Inset image: Representation of TN developed in Cytoscape software as described in Fig. 2, with 147 ‘target nodes’ (‘*HB* + *D*’, ‘*pure-driver*’, ‘*pure-HB*’) including 27 genes (deep grey circles) and categories (different colour codes) of 73 symptoms and 47 diseases, other than ‘target nodes’ (light grey) and all edges (grey). Pictorial image: The connections of only ‘target nodes’ including categories (big circles) of symptoms and diseases in TN, exhibiting details of nodes and edges: triangular (open) inter-links of three gene nodes (*ACTB*, *ADAR*, *CTNNB1*) and respective nodes of 12 symptoms and 11 diseases with same colour codes (for both nodes and edges); other nodes (white-coloured with grey border) without having triangular inter-links and their connections (grey-coloured edges). Both inset and pictorial images: Nodes are represented as different shapes (symptoms:diamond, diseases:rectangle, genes:circle) and sizes (connectivity scores adjusted by the ‘continuous mapping of node size’ in ranges between 25 and 60 pts.) with grey borders (illustrated with 2 pts.). The widths of the edges display respective metrics, which are adjusted by 0.5–2 pts. of ‘continuous mapping of edge width’ in the edge network style of the Cytoscape. (**b–e**) Heat maps: Representation of the pairwise SSS values (0–1 with colour codes) of ‘*target nodes*’ of TN including 73 symptoms (**b**), 47 diseases (**c**) and 27 genes (**d**, **e**). The pairwise SSS measurements (vide ‘Methodology’ section) are calculated as SSS-I for genes (**d**) and ‘SSS-II for symptoms (**b**), diseases (**c**) and genes (**e**). The columns of heatmaps (**b** and **c**) include codes (mentioned right side of each term in a row) of symptoms (**b**) and diseases (**c**). The vertical bars (left side of each heat map) demonstrate nodes having three topological properties (different colour codes) of centrality measurements and categories (same colour codes as in ‘inset in **a**’) of symptoms and diseases of the network. The summary of classifier ROC-AUC statistics (threshold scores, accuracy scores in %, AUC scores) of SSSs (**b**–**e**) are presented adjacent to the colour bar.

The stepwise construction (Fig. 2) of TN (network density: 0.029, average clustering coefficient:0.108) provided nodes (total 329) and their edges (total 3114) of 92 symptoms, 48 diseases and 189 genes (inset in Fig. 3a).

### Finding out the ‘target nodes under TN’ (TG-TN) for symptoms, diseases and genes

The topological assessment on TN evaluated 73 symptoms, 47 diseases and 27 genes (Fig. 3a) under three different properties namely ‘*both HB and driver’ (‘HB* + *D’)*, ‘*pure-driver*’, ‘*pure-HB*’ nodes. Further, the important nodes including 73 ‘target symptoms’ (‘*HB* + *D’*:*‘pure-driver*’:‘*pure-HB*’ = 16:44:13) and 47 ‘target diseases’ (‘*HB* + *D’*:*‘pure-driver*’:‘*pure-HB*’ = 8:0:39) were classified into respective six and eight different categories, respectively (Fig. 3a). The 27 TG-TN showed properties (Fig. 3d and e) of ‘*HB* + *D*’ (*CTNNB1*), ‘*pure-HB*’ (16 genes) and ‘pure-*driver*’ nodes (10 genes).

### Construction of ‘combined gene network’ (CGN) using selected co-expressed genes and TG-TN for COVID-19

#### Finding out the co-expressed genes and the formation of CGN

**Figure 4:**
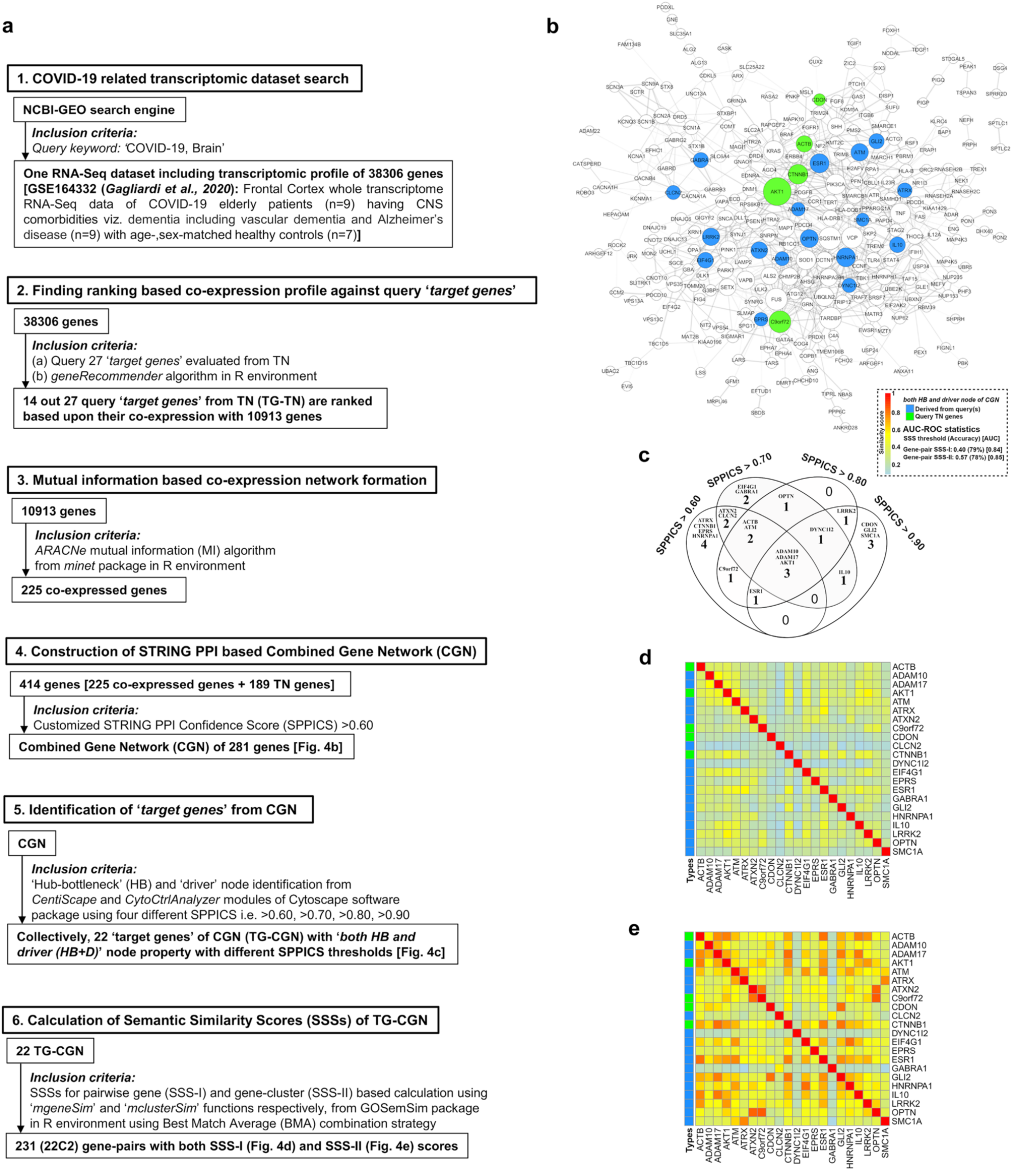
The model of ‘combined gene network’ (CGN) and its ‘*target nodes*’ with their pairwise semantic similarity scores (SSS) for COVID-19: (**a**) Study design for the construction of the CGN is represented stepwise (point 1–6). (**b**) The PPI interactome model of CGN is a continuous network consisting of total 281 nodes of gene products/proteins and 793 edges corresponding to the functional connectivities between nodes. The CGN is constructed using SPPICS > 0.60 as the widths of the edges, which are adjusted by 0.5 to 5 pts. of ‘continuous mapping of edge width’ in the edge network style of the Cytoscape. The colour nodes (inset) represent 22 ‘*target genes*’ having both ‘HB and driver’ properties including five ‘query genes’ derived from TN (green nodes) and 17 ‘co-expressed genes’ (dodger blue) derived from RNA-Seq data of COVID-19 patients. The other nodes of the CGN are kept white-coloured with grey-borders (illustrated with 2 pts.). The sizes of the nodes indicate their connectivities (higher the value, higher will be the size) adjusted by the ‘continuous mapping of node size’ in ranges between 25 and 60 pts. for the lowest and highest node size respectively. (**c**) The Venn diagrams indicate the changes in number of ‘*target genes*’ (both ‘HB and driver’) with their names derived from CGNs developed using multiple SPPICS thresholds including > 0.60, > 0.70, > 0.80 and > 0.90 for inclusion of all possible ‘*target genes*’ for better interpretations. (**d** and **e**) Heat maps represent the pairwise SSS values (0–1 with colour codes) of 22 ‘*target genes*’ of CGN. The values of SSS-I (**d**) and SSS-II (**e**) are direct and indirect associations (vide ‘Methodology’ section), respectively. All ‘*target genes*’ in CGN belong to ‘HB and driver’ topological properties of centrality measurements. The colours of the vertical bars (left side of each heat map) indicate types (vide inset) of ‘*target genes*’. The summary of classifier ROC-AUC statistics (threshold scores, accuracy scores in %, AUC scores) of SSSs is presented in adjacent to the colour bar.

Total 225 co-expressed genes were identified from RNA-Seq data and used for the construction of CGN along with 189 genes identified in TN. The stepwise construction (Fig. 4a) of CGN (network density: 0.010, average clustering coefficient:0.169) using a confidence score (SPPICS) > 0.6 provided 281 gene nodes (162 genes from TN including 27 TG-TN) and their 793 edges (Fig. 4b).

#### Evaluation of ‘target genes under CGN’ (TG-CGN)

Collectively, the 22 gene nodes found as ‘*HB* + *D*’ from the four different PPI-networks (CGNs) constructed using different confidence scores (SPPICS) viz. > 0.70, > 0.80 and > 0.90 and, were marked as TG-CGN for COVID-19 (Fig. 4c). Interestingly, five genes (*ACTB*, *AKT1*, *C9orf72*, *CDON*, *CTNNB1)* out of 22 TG-CGN were found to occur within 14 query genes (green-coloured nodes in Fig. 4b).

### Evaluation of pairwise values of SSS for ‘target nodes’

#### Finding out the pairwise SSS-II values of TG-TN for symptoms and diseases

The 73 symptoms (Fig. 3b) and 47 diseases (Fig. 3c) as TG-TN provided pairwise SSS-II scores for 2628 (^73^C_2_) and 1081 (^47^C_2_) combinations with their respective six and eight different categories. The 1196 symptom-pairs and 130 diseases-pairs appeared significant based on their respective cut-off values.

#### Finding out the pairwise values of SSS-I and SSS-II of ‘target genes’

The 27 TG-TN showed 351 (^27^C_2_) gene-pairs with SSS-I values (Fig. 3d), and provided 276 (^24^C_2_) gene-pairs with SSS-II values (Fig. 3e). The SSS-II calculation did not arise for three gene nodes (*ANG*, *ANXA*, *C9orf72*) because they did not have PPI connections in TN. The 22 TG-CGN genes exhibited 231 (^22^C_2_) gene-pairs with both SSS-I (Fig. 4d) and SSS-II values (Fig. 4e) indicating that the 22 genes had connections with other genes in CGN.

**Figure 5:**
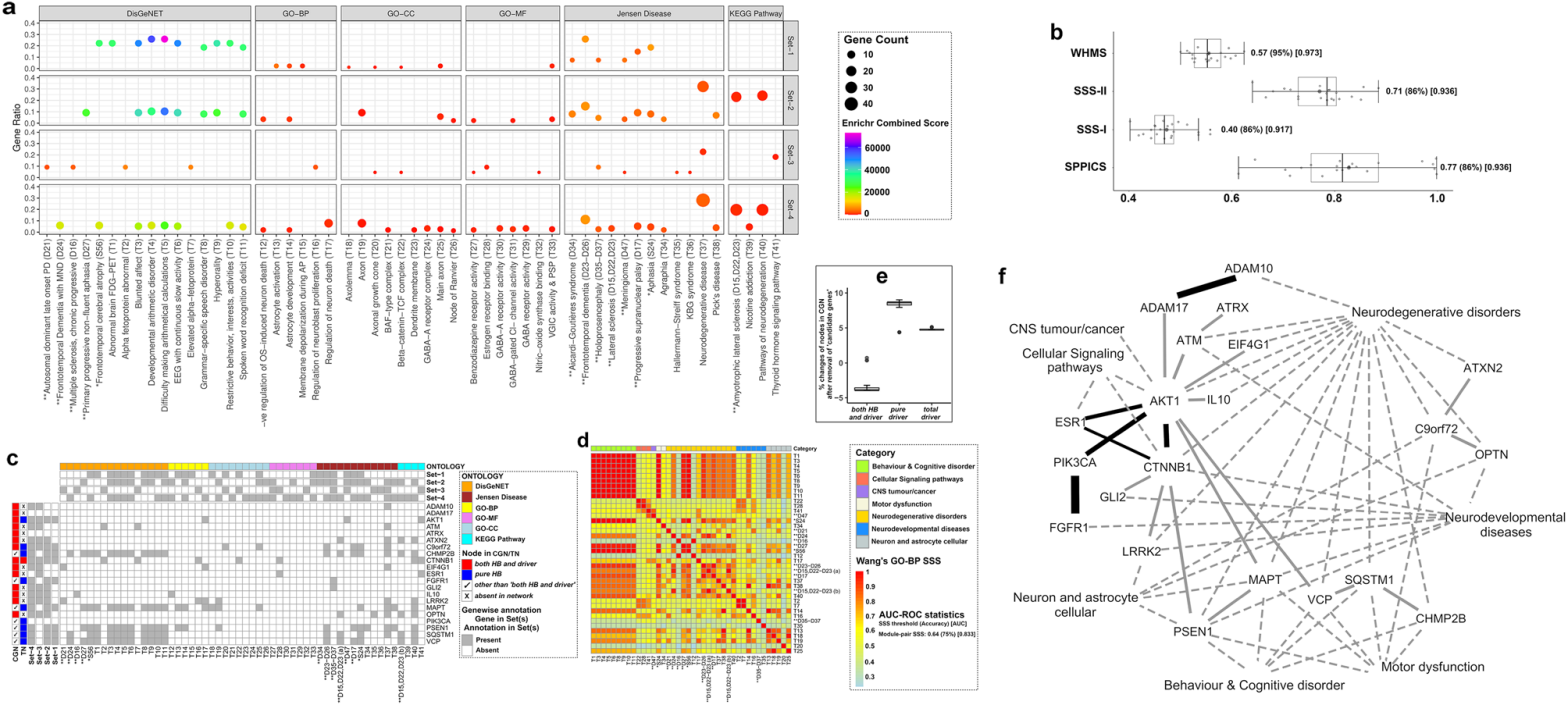
The ‘*candidate genes*’ and their functional modules for neurological manifestations of COVID-19: (**a**) Bubble plot: Results of Enrichment analysis against four sets of genes (‘*target genes*’ and their connected genes), exhibiting nervous system-specific functional annotations/terms (X-axis) across six resources, characterised by ‘gene count’ (size of bubbles adjusted with 0.5 to 4 pts.), ‘combined score’ (log(p-value) × z-value > 147 adjusted in VIBGYOR colour gradient of bubbles) and corresponding ‘gene ratio’ (Y-axis). (**b**) Box plot: Data (X-axis) of interaction score types (Y-axis) i.e., SSS-I, SSS-II, SPPICS and WHMS of ‘*candidate genes*’. (**c**) Checkerboard: ‘*Candidate genes*’ (Y-axis) with their status of topological properties in networks (vertical bars), associated with enriched 54 functional annotation terms (X-axis) evaluated under six different ontology categories (horizontal bar) manually curated from results of the bubble plot (**a**). (**d**) Heat map: Pairwise GO-BP SSS-II values (0.23–1.0 with colour codes) of 40 enriched terms with their categories (colours in horizontal bar), overrepresented with at least one ‘candidate gene’. (**e**) Box plot: Data of % changes (Y-axis) of topological properties (X-axis) in CGN using leave-one-out method by ‘*candidate genes*’ to cross-validate disease-causing ‘*indispensable*’ driver nodes in the network. (**f**) Pictorial presentation (developed in Cytoscape software): the interactome model of 21 ‘*candidate genes*’ and their associated functional seven categories of Enrichr terms related to neurological manifestations of COVID-19. Lines represent gene-pair interactions (solid lines: width as WHMS adjusted by 3–10 pts.) with the ‘*prevalent*’ links (dark solid lines) of ‘*candidate genes*’ having WHMS more than cut-off values (given in **b**) and functional links (dotted lines: width adjusted by 2 pts.) of genes with categories (given in **d**) of Enrichr terms. [Box plot (**b** and **e**) summary: Data with quartile values (edges of box), inter-quartile range, median value (black vertical line inside box), mean value (grey-coloured filled circle in box), maximum and minimum values (two vertical lines), and their out layers; Classifier statistics (ROC-AUC) summary: optimal threshold score(accuracy score)AUC score (vide: right side of box plots in b; adjacent to the colour bar in **d**); Codes of Enrichr annotation terms (**c** and **d**): ‘D15,D22,D23(a)’ and ‘D15,D22,D23(b)’ as ‘Lateral sclerosis’ and ‘Amyotrophic lateral sclerosis’ respectively.]

Five genes (green-coloured nodes in Fig. 4b), common in both networks (TN and CGN), showed their pairwise unique SSS-I (Figs. 3d and 4d) values irrespective of the networks. The gene-pairs of TGs with SSS-I (Figs. 3d and 4d) and SSS-II (Figs. 3e and 4e) scores above their respective cut-off values were selected as statistically significant once under both networks (TN and CGN). In TG-TN, 20 gene-pairs were common among selected gene-pairs having significant SSS-I (31 gene-pairs, Fig. 3d) and SSS-II (81 gene-pairs, Fig. 3d) values. In CGN, 33 gene-pairs were common among selected gene-pairs having significant SSS-I (33 gene-pairs, Fig. 4d) and SSS-II (143 gene-pairs, Fig. 4e) values. Among the statistically selected common gene-pairs of TGs, nine in TN and 13 in CGN showed physical PPI interactions with SPPICS values (Table 2) in their respective networks. Interestingly, the PPI link viz. *AKT1-CTNNB1* was evident in both TN and CGN (Figs. 3a and 4b, Table 2). Therefore, 21 gene-pairs (eight from TN, 12 from CGN, one common for both networks) exhibiting SSS-I, SSS-II and SPPICS values, were designated as *‘candidate genes’* pairs for COVID-19 (Fig. 5f).

**Table 2:** Summary of pairwise ‘*candidate genes*’ with their properties evaluated as ‘*prevalent*’ and ‘*non-prevalent*’ characters and their functional links having statuses with interaction scores vide SSS-I, SSS-II, SPPICS and WHMS values.

S. no	Gene-pair	SSS-I Value (Property)	SSS-II Value (Property)	SPPICS Value (Property)	WHMS Value
**Prevalent**					
1	*FGFR1-PIK3CA*	(0.54) Is_a	(0.81) Is_a	(0.97) Strong	0.62
2	*ADAM10-ADAM17*	(0.56) Is_a	(0.77) Is_a	(0.86) Strong	0.61
3	*AKT1-CTNNB1*	(0.49) Is_a	(0.79) Is_a	(0.99) Strong	0.60
4	*AKT1-PIK3CA*	(0.48) Is_a	(0.79) Is_a	(0.99) Strong	0.59
5	*AKT1-ESR1*	(0.47) Is_a	(0.80) Is_a	(0.99) Strong	0.59
6	*CTNNB1-ESR1*	(0.48) Is_a	(0.83) Is_a	(0.84) Strong	0.58
**Non-prevalent**					
1	*AKT1-ATM*	(0.54) Is_a	(0.71) Is_a	(0.80) Strong	0.57
2	*CTNNB1-PSEN1*	(0.47) Is_a	(0.68) Part_of	(0.99) Strong	0.56
3	*SQSTM1-VCP*	(0.51) Is_a	(0.79) Is_a	(0.70) Weak	0.56
4	*ADAM17-AKT1*	(0.50) Is_a	(0.78) Is_a	(0.74) Weak	0.55
5	*AKT1-IL10*	(0.46) Is_a	(0.77) Is_a	(0.83) Strong	0.55
6	*CHMP2B-SQSTM1*	(0.48) Is_a	(0.73) Is_a	(0.81) Strong	0.55
7	*C9orf72-OPTN*	(0.43) Is_a	(0.89) Is_a	(0.82) Strong	0.55
8	*AKT1-MAPT*	(0.45) Is_a	(0.73) Is_a	(0.80) Strong	0.53
9	*CTNNB1-LRRK2*	(0.44) Is_a	(0.70) Part_of	(0.83) Strong	0.53
10	*MAPT-PSEN1*	(0.45) Is_a	(0.64) Part_of	(0.89) Strong	0.53
11	*AKT1-VCP*	(0.42) Is_a	(0.80) Is_a	(0.78) Strong	0.52
12	*ATM-ATRX*	(0.44) Is_a	(0.79) Is_a	(0.71) Weak	0.52
13	*C9orf72-ATXN2*	(0.40) Is_a	(0.86) Is_a	(0.75) Weak	0.52
14	*CTNNB1-GLI2*	(0.45) Is_a	(0.85) Is_a	(0.61) Weak	0.51
15	*AKT1-EIF4G1*	(0.48) Is_a	(0.67) Part_of	(0.65) Weak	0.50

The *prevalent* pairwise ‘*candidate genes*’ are selected based on values of gene-pairs more than the cut-off value of WHMS viz. 0.57 (vide Fig. 5b and f). The terms ‘Is_a’ (subtype) and ‘Part_of’’ (component) represent the functional associations of GO-based terms hierarchically (ancestors-descendants relationship) organised in directed acyclic graph (DAG). The terms ‘Is_a’ and ‘Part_of’ properties are considered with the gene-pairs having scores greater than and less than, respectively the cut-off values of SSS-I (viz. 0.40) and SSS-II (viz. 0.71). The terms ‘Strong’ and ‘Weak’ represent the link strength having values of SPPICS to gene-pairs with scores greater than and less than, respectively the cut-off value (viz. 0.77). The cut-off values (optimal threshold score of ROC) are given in Fig. 5b.

### Selection and categorisation of ‘candidate gene’ *pairs* by the formulation of WHMS

The selected 21 ‘*candidate genes*’ with their pairwise 21 interactions showed excellent (AUC > 0.90) classifications based on interaction values of SSS-I, SSS-II and SPPICS. The interaction values showed the same accuracy score of 0.86, and this score was used as weightage for respective case to calculate integrated WHMS of interactions of the ‘*candidate genes*’. Considering statistical accuracy (95%) and excellent classification with the highest AUC value (0.97), the values of WHMS of ‘*candidate genes*’ pairs were considered a better choice for further analysis (Fig. 5b and Table 2). The six pairwise interactions (*ADAM10-ADAM17*, *AKT1-CTNNB1*, *AKT1-ESR1*, *AKT1-PIK3CA*, *CTNNB1-ESR1*, *FGFR1-PIK3CA*) of ‘*candidate genes*’ showed WHMS values (Table 2) greater than the cut-off value (0.57) for WHMS (Fig. 5b) and were considered as *prevalent* pairs-wise interactions of the ‘*candidate genes*’ that were associated with functional aspects in COVID-19 (dark solid edges in Fig. 5f). Notably, these interactions of *prevalent* ‘*candidate genes*’ had values of SSS-I, SSS-II and SPPICS greater than their respective cut-off values (Fig. 5b and Table 2). The remaining 15 pairwise interactions of ‘*candidate genes*’ (non-*prevalent*) showed WHMS values below the cut-off value (Table 2, light solid edges in Fig. 5f). Few of them exhibited values of SSS-I, SSS-II and SPPICS below the respective cut-off values. Notably, 21 pairwise ‘*candidate genes*’ presented SSS-I values greater than the cut-off (0.40) value (Table 2).

### Enrichr analysis of ‘*target genes*’ and Integration of annotation terms

Enrichr annotation of four sets of genes with ‘*target genes*’ (set-1 for TG-TN and set-3 for TG-CGN) and their connected genes (set-2 and set-4), gave off statistically significant a total of 159 terms (data not shown) including several common terms among four sets. The statistically significant (FDR-adjusted p-value < 0.05) Enrichr annotation terms were further cross-validated manually with selected (above cut-off values of SSS-II) ‘*target nodes*’ of the symptoms (Fig. 3b) and diseases (Fig. 3c) found in TN analysis. 13 terms (two symptoms,11 diseases) were common (Fig. 5a) among annotations under (i) DisGeNET (four diseases, one symptom), (ii) Jensen disease (six diseases, one symptom) (iii) KEGG pathway (one disease). The rest of the total terms showed selected 41 terms (Fig. 5a) associated with functional annotations of the nervous system, including 11 in DisGeNET (T1-T11), six in GO-BP (T12-T17), nine in GO-CC (T18-T26), seven in GO-MF (T27-T33), five in Jensen Disease (T34-T38) and three in KEGG pathway (T39-T41). Therefore, 54 (13 + 41) selected terms were implied to discover their functional links with ‘*candidate genes*’ in subsequent analysis.

### Finding out the functional annotations of ‘*candidate genes*’ and their categorisation

The selected 40 functional annotation terms against 21 ‘*candidate genes*’ (Fig. 5c), provided 780 pairwise (^40^C_2_) SSS-II values (Fig. 5d) and showed an accurate (75%) and good classification (AUC:0.83) with 330 SSS-II values above the cut-off value (0.64). These terms were categorised in to seven different functional modules (‘Behaviour & Cognitive disorder’, ‘Cellular Signaling pathways’, ‘CNS tumour/cancer’, ‘Motor dysfunction’, ‘Neurodegenerative disorders’, ‘Neurodevelopmental diseases’, ‘Neuron and astrocyte cellular’), comparing with functional modules categorised for symptoms and diseases in TN (Figs. 3b and 3c). Interestingly, six pairwise terms appeared to be common among statistically significant terms under (a) enriched for ‘*candidate genes*’ (Fig. 5d) and (b) ‘*target nodes*’ for symptoms/diseases under TN (Figs. 3b, 3c) and, presented greater (% more) pairwise SSS-II values with ‘*candidate genes*’ viz. atrophy-aphasia (3.7%) as symptom-pair and other five disease-pairs including (a) ‘progressive non-fluent aphasia’ with ‘classic progressive supranuclear palsy syndrome’ (15%), ‘amyotrophic lateral sclerosis’ (32.5%), ‘frontotemporal dementia’ (17.7%) and (b) ‘frontotemporal dementia’ with ‘amyotrophic lateral sclerosis’ (8.6%) and ‘progressive supranuclear palsy syndrome’ (17.2%).

### Finding out the essentiality of ‘*candidate genes*’

Cross-validation (leave-one-out method) of 21 ‘*candidate genes*’ resulted in a 3.15% average reduction in ‘*HB* + *D*’ node and average increases in 7.96% and 4.81% of ‘*pure-driver*’ and ‘total *driver*’ nodes respectively, in CGN network (Fig. 5e). The results signified the 21 ‘*candidate genes*’ as ‘*indispensable*’ driver nodes that were practically regulatory genes associated with neurological diseases in COVID-19.

### Assimilation of ‘*candidate genes*’ with functional annotations to develop brain-related functional modules

The 21 ‘*candidate genes*’, their 21 interactions having WHMS (Table 2) and links with corresponding functional annotations (Fig. 5c) under different categories (Fig. 5d) are represented in a pictorial diagram (Fig. 5f) for better interpretations. The seven *prevalent ‘candidate genes*’ (*ADAM10*, *ADAM17*, *AKT1*, *CTNNB1*, *ESR1*, *FGFR1*, *PIK3CA*) showed direct associations with enriched terms under the categories of ‘Neurodegenerative disorders’, ‘Neurodevelopmental diseases’, ‘CNS tumour/cancer’ and ‘Cellular Signaling pathways’. Their indirect associations with enriched terms under the categories of ‘Neuron and astrocyte cellular’ events, ‘Behaviour & Cognitive disorder’ and ‘Motor dysfunction’ appeared to have interactions with *non-prevalent* ‘*candidate genes*’ (Fig. 5f).

## Discussion

The present study is a novel approach of integrated network-based multiple computational analyses of two networks, viz. TN and CGN to find the ‘*disease-related regulatory genes*’ associated with functional (transcriptional and translational) cellular entities necessary for understanding the molecular basis of brain pathophysiological phenotypes of COVID-19. To achieve the goal, we proceeded with the most robust approaches through multiple screening steps including (a) finding the two sets of predictive ‘*target genes*’, evaluated from TN and CGN with their PPIs having STRING ‘*combined scores*’ (SPPICS) as priori analysis, (b) evaluation of functional associations by ‘semantic similarity scores’ (SSS) of two sets of ‘*target genes*’, (c) screening ‘*target genes*’ by cumulating PPIs having both STRING-CS and SSS by selection with given threshold values for respective PPI scores to find ‘*candidate genes*’, (d) formulating integrated scores (WHMS) combining SPPICS and SSS for giving weightage to PPIs of ‘*candidate genes*’ for further categorisation, (e) assimilation of ‘*annotation terms*’ (symptoms/diseases) with genes among ‘*candidate nodes*’ through posteriori enrichment analysis to get functional module. Notably, ‘*target nodes*’ for symptoms and diseases evaluated from TN were manually curated and integrated with suitable Enrichr annotations for better interpretation. The classification statistics (ROC-AUC) and cut-off values (optimal thresholds for ROC) identified the PPIs with their association scores (SPPICS, SSS, WHMS) for the respective steps most accurately (AUC > 0.8) with minimum false positive interpretation. Furthermore, all 21 ‘*candidate genes*’ appeared co-expressive. They became almost equally ‘*indispensable*’ after screening for their controllability property on CGN. Finally, the ‘*candidate genes*’ were categorised based on pairwise analysis of values of WHMS, SSS and SPPICS to find *prevalent* vs. *non-prevalent* ‘*candidate genes*’ with their pattern (‘*is_a*’ vs ‘*part_of*’) of relationship with neurological manifestations in COVID-19. The pathophysiological relevance of *prevalent* ‘*candidate genes*’ with COVID-19 has been discussed thoroughly.

In our study, two networks (TN (Fig. 3a) and CGN (Fig. 4b)) were analysed to find the ‘*target nodes*’, which satisfied the three properties, viz. ‘*hub*’, ‘*bottlenecks*’ and ‘*driver*’ together for COVID-19. Separate studies indicate that host proteins targeted by viral proteins show the node properties of hubs and high-betweenness centrality^25^ and, ‘*indispensable*’ driver controllability^25,26^ in a host protein network. The ‘*target genes*’ (TG) evaluated from TN (TG-TN) showed node properties of hub-bottleneck (HB or ‘*date-hubs*’ i.e., together hub and bottlenecks), driver and both (HB and driver) (Fig. 3d and e). All ‘*target genes*’ (TG) evaluated from CGN (TG-CGN) showed node properties as both HB and driver (Fig. 4b and c). In fact, the number of driver nodes compared to the driver nodes themselves appears crucial for maintenance of the controllability of a network^25,26^. In our study, the finally selected 21 ‘*candidate genes*’ appeared to be ‘*indispensable*’ as the number of driver nodes increased (4.81% for total drivers, 7.96% for drivers but non-hub-bottlenecks i.e., ‘*pure-drivers*’) in the CGN after removal of one of the ‘*candidate genes*’ (Fig. 5e).

Next, the SPPICS were applied to construct possible PPI connections of new genes in TN (Fig. 3a) and CGN (Fig. 4b) networks related to the brain in COVID-19. The SPPICS provides quantitative measurement of physical and functional PPI evidence derived from available online resources. It lacks experimental evidences of functional entities related to regulatory mechanism in physiological context of cells, as part of its calculation. The Gene Ontology resources provide a model of hierarchically (ancestors-descendants relationship) organised directed acyclic graph (DAG) having GO-terms as nodes and functional association as directed edges within each hierarchy by ‘*is_a*’ (subtype) and ‘*part_of*’ (component) relationships associated with gene/protein functionality (molecular function, cellular component and biological process) description. GO-based biological process (GO-BP) provides cohesive evidences on protein interactions, related to both physical and functional networks of molecular events in cellular physiology^27^. The ‘*candidate genes*’ for a disease show common biological pathway(s)^18^. Therefore, in our study, the functional associations among ‘*target nodes*’ were analysed by semantic comparison of GO-BP annotations quantitatively through computing similarities between gene-pairs (SSS-I measurement) (Figs. 3d and 4d) and clustering gene/symptoms/disease/module-pairs (SSS-II measurement) into known pathways (Figs. 3b, c, e, 4e and 5d).

The conventional SSS-I provided pairwise ‘*direct association*’ based on comparative assessment of associated GO-BP terms of two ‘*target genes*’ (Figs. 3d and 4d). It has been reported that the genes and their functionally connected co-expressive genes show tissue-specific expressions and regulations, and exhibit pleiotropic effects, i.e., sharing common symptoms and diseases^29,30^. Based on this concept, the estimation of SSS-II values was newly introduced in our study (Figs. 3e and 4e). The SSS-II values provided pairwise ‘*indirect association*’ based on the summated contribution of comparative assessment of associated GO-BP terms of gene-clusters (connected genes) against targeted gene-pairs. Our data indicated that the classification of both SSS-I and SSS-II values were statistically robust (AUC: 0.91 and 0.93) with the different range of values and had respective accurate (0.40 and 0.71) threshold values for ROC to interpret the results most stringently (Fig. 5b). Interestingly, the gene-pairs found as common PPI in TN and CGN networks showed the same values of SSS-I whereas SSS-II values varied for networks. For example, *CTNNB1*-*AKT1* gene-pair among ‘*target genes*’, found as common PPI in both TN (Fig. 3a) and CGN (Fig. 4b), showed equal SSS-I value (0.487) (Figs. 3d and 4d). The SSS-II values of this gene-pair varied for TN (0.783) and CGN (0.805) (Figs. 3e and 4e). Additionally, certain gene-pairs having considerable (above a threshold value) SSS-I values appeared to have low (below a threshold value) or zero (‘null functional similarity’) SSS-II values, including *AKT1*-*FGFR1* (SSS-I: 0.485—above threshold; (Fig. 3d), SSS-II: 0.69—below the threshold; (Fig. 3e)), *C9orf72*-*SQSTM1* (SSS-I: 0.462—above threshold; (Fig. 3d), SSS-II: 0; (Fig. 3e)). Therefore, the gene-pairs with significant values of both SSS-I and SSS-II were considered for better interpretation of the results in our study.

Irrespective of the network, the SSS-I values of gene-pairs/PPIs might depict the global and existing ‘*is_a*’ and/or ‘*part_of*’ semantic similarity available in the GO-BP annotation data and therefore would remain the same for representing generalised pathophysiological functions for any disease condition. Alternatively, the SSS-II values for gene-pairs varied due to different constituents in ‘gene-clusters’, which provided the ‘*is_a*’ and/or ‘*part_of*’ functional relationship by sharing common GO-BP annotation terms to reflect the discrete or pleiotropic effects of genes among networks (Table 2). Particularly, the zero value of SSS-II of a gene-pair indicated that ‘*gene-clusters*’ (connected genes) against the gene-pair had not been well-supported by current literature-based evidences related to COVID-19 neurological symptoms. Therefore, the SSS-II values might provide a better disease-specific metric for the event of disassembly in the homeostatic genetic connectivity that gets perturbed during COVID-19 insult.

The better quality of the PPI network improves the prediction accuracy to determine the ‘*candidate genes*’ for a disease. The STRING database comprises genes from prior knowledge and thereby provides a PPI model with certain limitations. The SSS-based PPI network includes genes having sufficient annotation information and so has GO annotation biasness. The integration of two scores, viz. SPPICS of STRING-based PPI network and SSS of anatomy-based gene network by introducing ‘*accuracy values of ROC*’ as weightage given to the respective scores followed by summation of them, is reported to develop the better quality of network by filtering out the false positive interactions^28^. In our study, the same principle of weightage (‘*accuracy value of ROC*’) was applied to evaluate the weighted scores of SPPICS, SSS-I, SSS-II followed by calculating their harmonic mean in order to evolve the integrated scores (WHMS) for those gene-pairs which satisfied the criteria of having (a) three individual scores (SPPICS, SSS-I, SSS-II) and (b) at least one score with value above respective threshold level (Fig. 5b). The integrated scores of total 21 gene-pairs showed statistically strong fitted (AUC > 0.9) and most accurate (95%) interactions (Fig. 5b and solid edges in Fig. 5f), and provided 21 ‘*candidate genes*’ (Fig. 5c and f) associated with neurological insults (Fig. 5f) in COVID-19. All 21 ‘candidate genes’ (Fig. 5c) appeared to be derived from RNA-Seq data (Fig. 4b) and thus considered as co-expressed genes of COVID-19 in the brain.

All 21 gene-pairs/PPIs of ‘*candidate genes*’ showed SSS-I values (Figs. 3d, 4d, vide Point 4.2 in the results section) above the respective threshold value and therefore represented as ‘*is_a*’ functional relationship (Table 2) in the semantic similarity of GO-BP annotations for generalised pathophysiological functions irrespective of disease. Based on the threshold value of integrated PPI scores (WHMS), 21 pairwise ‘*candidate genes*’ were classified as ‘*prevalent*’ and ‘*non-prevalent*’ ‘*candidate genes*’ (Table 2). Six pairs of seven ‘*prevalent*’ ‘*candidate genes*’ showed strong database-dependent putative interaction scores (SPPICS) (Figs. 3a and 4b) and subsequently satisfied SSS-II values (Figs. 3e and 4e) above the threshold levels representing ‘*is_a*’ relationship (Table 2) with neuro-pathological manifestations in COVID-19. The ‘*non-prevalent*’ ‘*candidate genes*’ found to have varied SPPICS scores (strong and weak) and different relationships (‘*is_a*’ and ‘*part-of*’) among their gene-pairs (Table 2). The ‘*prevalent*’ ‘*candidate genes*’ (*ADAM10*, *ADAM17*, *AKT1*, *CTNNB1*, *ESR1*, *FGFR1*, *PIK3CA*) might have the most prominent pathophysiological relevance in COVID-19.

The pathophysiological action of SARS-CoV-2 in brain tissue cells begins with its binding to ACE2 receptors of the cell membrane. After viral endocytosis is over, ADAM17 directs the shedding of the ectodomain of the receptors^31^ and enhances the formation of TNF-α leading to escalation of the cytokine storm^1^. Dysfunction of ADAMs can also exacerbate Alzheimer’s disease condition through the misfolded Aβ pathology^32^, ischaemic stroke^33^ and vascular thrombosis^34^ via ACE2 and TNF-α receptors. Recently, *ADAM10* and *ADAM17* have been marked as the risk factors for cerebral infarction and hippocampal sclerosis related epilepsy^35^, respectively. In diabetic patients, an elevated activity of ADAM17 is found to enhance COVID-19 susceptibility^36^ through the AKT1*-*mediated pathway.

*AKT1* encodes protein kinase B, which is a part of the PI3K-NFκβ signalling pathway, involved in aberrant expression of IL10 and inflammation in severe coronavirus infection^32^. AKT1 can induce tumour formation through the upregulation of RNA binding protein EIF4G1^37^, coronavirus exit from endosomes via valosin-containing protein VCP^38^ and MAPT-associated tau protein formation in dementia-like cognitive impairment^39^. The altered AKT1-signalling pathway is also evident in ATM-associated autism spectrum disorders that may exaggerate COVID-19^40^.

*CTNNB1* expresses β-catenin related to the Wnt-signalling pathway and gets downregulated in COVID-19^41^ through the activation of glycogen synthase kinase 3β in the prefrontal cortex and dorsal hippocampus^42^. Defects in the formation of β-catenin cause disruption of the blood–brain barrier^43^ leading to the development of cerebrovascular thrombosis^44^, headache^45^, stroke^46^ and epileptic seizure^47^ during or in the aftermath of COVID-19. Stress-induced Dickkopf-1 protein formation prevents *CTNNB1* gene function in the hippocampus, thereby impairing memory^48^. Uncontrolled interactions of CTNNB1 with PSEN1^49^ and GLI2^50^ are linked to skin tumorigenesis, which may be suggestive for their possible involvement in COVID-19. Moreover, abnormalities in PSEN1^51^ and GLI2^52^ functions, associated with the *CTNNB1* gene are likely to be implicated in developing Alzheimer’s disease- and holoprosencephaly-like features in COVID-19.

*ESR1* gene encodes estrogen receptor 1 that occurs primarily in the medial preoptic area and ventromedial nucleus of the hypothalamus, which regulates diverse reproductive functions of both males and females^53^. ESR1 deems to share CTNNB1-^54^ and AKT1-^55^ mediated signalling pathways to accelerate cancer and neurodegeneration, respectively. Moreover, estrogen inhibits inflammation and immune responses in COVID-19 and reduces the COVID-19 susceptibility in females than in males, because of its higher concentration and a greater number of ESR1 receptors in target tissues^56^.

In the adult brain, the *PIK3CA* gene product PI3K via the AKT1-pathway may exaggerate neurodegeneration in Alzheimer’s disease^57^, and FGFR1 dysregulation leads to ischemic stroke^58^ and holoprosencephaly^59^. Moreover, synchronised *PIK3CA* mutation and FGFR1 alteration are associated with *ESR1*-positive breast cancer^60^. Since COVID-19 develops inflammatory burst and lymphopenia, SARS-CoV-2-associated illness therefore may aggravate cancer prognosis^59^.

Notably, two *prevalent* genes *CTNNB1* and *AKT1* appeared to be common for both TN (Fig. 3a) and CGN (Fig. 4b). Both genes showed SSS-II (network-specific semantic similarity score) values greater than threshold values in respective cases (Figs. 3e and 4e), and therefore functionally interlinked (Fig. 5f). *CTNNB1* appeared as the lone gene having both HB and driver node properties in TN. Interestingly, *CTNNB1* was the only gene which formed a ‘tripartite open network’ that linked with eight symptoms and those symptoms remained connected with eight diseases (Fig. 3a). *CTNNB1* in TN got connections with (a) five symptoms (viz. cerebral ischemia, vascular thrombosis, intracranial hypertension, seizures and epileptic seizures) in the central nervous system (CNS), (b) two symptoms (viz. hypertonia and fatigue) in the peripheral nervous system (PNS) and (c) one psychiatric symptom (viz. behavioral disorder). Moreover, it demonstrated that three symptoms connected with *CTNNB1* in the present tripartite network, also happened to occur in other diseases, coinfected with COVID-19, viz. (a) cerebral ischemia in alobar, lobar and semilobar holoprosencephaly, Behçet disease, early infantile epileptic encephalopathy, MELAS and meningioma; (b) vascular thrombosis in alobar, lobar and semilobar holoprosencephaly, amyotrophic lateral sclerosis and MELAS; (c) intracranial hypertension in MELAS. But no data is available yet about the rest of the five symptoms in any other diseases challenged none-ever with SARS-CoV-2. This suggests that certain neurological symptoms of COVID-19 are intermingled with other diseases and need special clinical attention.

In conclusion, the present study, however, suffers from two limitations regarding the (a) status of COVID-19 patients who had mixed implications of neurological symptoms/manifestations during hospitalisation in most cases, long-term reports in few cases and without having any detail in other cases as reported in the literature (Table 1), and (b) use of a small cohort of a transcriptomic dataset of patients having SARS-CoV-2 viruses in brain autopsy samples^23^, available only at the time of study period.

## Supplementary Information

Supplementary Tables.

Supplementary Information 2.

## Data Availability

All data are available in the paper.
