# Supplementary material for: Integrated network-based multiple computational analyses for identification of co-expressed candidate genes associated with neurological manifestations of COVID-19
Source: Sci Rep. 2022 Oct 13;12:17141. doi: 10.1038/s41598-022-21109-3 (PMC9558001; doi:10.1038/s41598-022-21109-3)
Supplement: Supplementary file 2 — Supplementary Information 2. [file 41598_2022_21109_MOESM2_ESM.pdf]

Supplementary File 2: Details of the methodological resources (software, tools) of the bioinformatics/computation analyses the present study. The table presents list of web links mentioned in “Methods” section with reference number of original articles cited in the web links. The list of original articles cited in respective web links are listed below of the table.

**Database/Tool**	**Available at**	**References**
PubMed	https://pubmed.ncbi.nlm.nih.gov/	Well-documented
HPO	https://hpo.jax.org/	[1]
MalaCards	https://www.malacards.org/	[2]
STRING	https://string-db.org/	[3]
Cytoscape	http://www.cytoscape.org/	[4]
geneRecommender	https://www.bioconductor.org/packages/release/bioc/html/geneRecommender.html	[5]
minet	https://www.bioconductor.org/packages/release/bioc/html/minet.html	[6]
ARACNe	https://rdrr.io/bioc/minet/man/aracne.html	[7]
CentiScaPe	http://chianti.ucsd.edu/cyto_web/plugins/index.php	[8]
CytoCtrlAnalyser	https://apps.cytoscape.org/apps/cytoctrlanalyser	[9]
GOSemSim	http://bioconductor.org/packages/release/bioc/html/GOSemSim.html	[10]
mgeneSim	https://rdrr.io/bioc/GOSemSim/man/mgeneSim.html	[11]
mclusterSim	https://rdrr.io/bioc/GOSemSim/man/mclusterSim.html	[11]
pROC	https://cran.r-project.org/web/packages/pROC/index.html	[11]
KEGG pathway	https://www.genome.jp/kegg/pathway.html	[12]
Enrichr	https://maayanlab.cloud/Enrichr/	[13]

References

1. Köhler, S. *et al.* The Human Phenotype Ontology in 2021. *Nucleic Acids Res.* **49**, D1207-D1217; https://doi.org/10.1093/nar/gkaa1043 (2021).
2. Rappaport, N. *et al.* MalaCards: an amalgamated human disease compendium with diverse clinical and genetic annotation and structured search. *Nucleic Acids Res.* **45**, D877-D887; https://doi.org/10.1093/nar/gkw1012 (2017).
3. Szklarczyk, D. *et al.* The STRING database in 2021: customizable protein-protein networks, and functional characterization of user-uploaded gene/measurement sets. *Nucleic Acids Res.* **49**, D605-D612; https://doi.org/10.1093/nar/gkaa1074 (2021).
4. Shannon, P. *et al.* Cytoscape: a software environment for integrated models of biomolecular interaction networks. *Genome Res.* **13**, 2498-2504; https://doi.org/10.1101/gr.1239303 (2003).
5. Owen, A. B. *et al.* A gene recommender algorithm to identify coexpressed genes in C. elegans. *Genome Res.* **13**, 1828–1837; https://doi.org/10.1101/gr.1125403 (2003).
6. Meyer, P. E. *et al.* minet: A R/Bioconductor package for inferring large transcriptional networks using mutual information. *BMC Bioinform.* **9**, 461; https://doi.org/10.1186/1471-2105-9-461 (2008).
7. Margolin, A. A. *et al.* ARACNE: an algorithm for the reconstruction of gene regulatory networks in a mammalian cellular context. *BMC Bioinform.* **7**, S7; https://doi.org/10.1186/1471-2105-7-S1-S7 (2006).
8. Scardoni, G. *et al.* Analyzing biological network parameters with CentiScaPe. *Bioinformatics (Oxford, England)* **25**, 2857–2859; https://doi.org/10.1093/bioinformatics/btp517 (2009).
9. Wu, L. *et al.* CytoCtrlAnalyser: a Cytoscape app for biomolecular network controllability analysis. *Bioinformatics (Oxford, England)* **34**, 1428-1430; https://doi.org/10.1093/bioinformatics/btx764 (2018).
10. Yu, G. *et al.* GOSemSim: an R package for measuring semantic similarity among GO terms and gene products. *Bioinformatics (Oxford, England)* **26**, 976-978; https://doi.org/10.1093/bioinformatics/btq064 (2010).
11. Robin, X. *et al.* pROC: an open-source package for R and S+ to analyze and compare ROC curves. *BMC Bioinform.* **12**, 77; https://doi.org/10.1186/1471-2105-12-77 (2011).
12. Kanehisa, M., & Goto, S. KEGG: kyoto encyclopedia of genes and genomes. *Nucleic Acids Res.* **28**, 27–30; https://doi.org/10.1093/nar/28.1.27 (2000).
13. Xie, Z. *et al.* Gene Set Knowledge Discovery with Enrichr. *Current Protoc.* **1**, e90; https://doi.org/10.1002/cpz1.90 (2021).
